# AI-generated draft replies to patient messages: exploring effects of implementation

**DOI:** 10.3389/fdgth.2025.1588143

**Published:** 2025-06-12

**Authors:** Charlotte M. H. H. T. Bootsma-Robroeks, Jessica D. Workum, Stephanie C. E. Schuit, Anne Hoekman, Tarannom Mehri, Job N. Doornberg, Tom P. van der Laan, Rosanne C. Schoonbeek

**Affiliations:** ^1^ Department of Health Information Office, Information Management Healthcare, University Medical Center, Groningen, Netherlands; ^2^ Department of Pediatrics, Pediatrics Nephrology, Beatrix Children’s Hospital, University Medical Center, Groningen, Netherlands; ^3^ Department of Intensive Care, Elisabeth-TweeSteden Hospital, Tilburg, Netherlands; ^4^ Department of Adult Intensive Care, Erasmus MC University Medical Center, Rotterdam, Netherlands; ^5^ University Medical Center, Groningen, Netherlands; ^6^ Department of Trauma Surgery/Orthopedics, University Medical Center, Groningen, Netherlands; ^7^ Department of Orthopaedic Trauma, Flinders University Medical Center, Adelaide, SA, Australia; ^8^ Department of Otolaryngology—Head and Neck Surgery, University Medical Center, Groningen, Netherlands

**Keywords:** large language model (LLM), inbasket messages, adoption, time saving, LLM generated draft responses, electronic health records

## Abstract

**Introduction:**

The integration of Large Language Models (LLMs) in Electronic Health Records (EHRs) has the potential to reduce administrative burden. Validating these tools in real-world clinical settings is essential for responsible implementation. In this study, the effect of implementing LLM-generated draft responses to patient questions in our EHR is evaluated with regard to adoption, use and potential time savings.

**Material and methods:**

Physicians across 14 medical specialties in a non-English large academic hospital were invited to use LLM-generated draft replies during this prospective observational clinical cohort study of 16 weeks, choosing either the drafted or a blank reply. The adoption rate, the level of adjustments to the initial drafted responses compared to the final sent messages (using ROUGE-1 and BLEU-1 natural language processing scores), and the time spent on these adjustments were analyzed.

**Results:**

A total of 919 messages by 100 physicians were evaluated. Clinicians used the LLM draft in 58% of replies. Of these, 43% used a large part of the suggested text for the final answer (≥10% match drafted responses: ROUGE-1: 86% similarity, vs. blank replies: ROUGE-1: 16%). Total response time did not significantly different when using a blank reply compared to using a drafted reply with ≥10% match (157 vs. 153 s, *p* = 0.69).

**Discussion:**

General adoption of LLM-generated draft responses to patient messages was 58%, although the level of adjustments on the drafted message varied widely between medical specialties. This implicates safe use in a non-English, tertiary setting. The current implementation has not yet resulted in time savings, but a learning curve can be expected.

**Registration number:**

19035.

## Introduction

The rise of digital communication in healthcare has led to a significant increase in patient-initiated messages through electronic health record (EHR) portals, with clinicians interacting with up to 20 messages and spending 50 min in inbox a day, 37% being outside of working hours (1, 2). With the growing use of patient portals, this number is expected to increase over time. This surge in digital communication contributes to the increased administrative workload of the clinician, which may negatively affect their ability to deliver high quality care, job satisfaction and may contribute to potential burnout (3, 4).

The integration of Large Language Models (LLMs) in clinical settings is progressing swiftly, providing possible solutions for the increased administrative workload (5–7). LLMs have shown vast potential in summarizing clinical notes (8). Additionally, LLMs have been used in drafting responses to patient messages, but the adoption rate among clinicians was low (20%) and there were no notable time savings (9–11). However, detailed comparisons between draft and final replies remain unexplored, leaving adoption and time-saving factors unclear. There is a notable gap in research regarding the performance and utility of LLMs in real world clinical practice, as only 5% of LLM applications currently published in medical literature are evaluated with real patient care data (12). As most applications are explored within the United States, this gap is even more profound in non-English clinical settings (13). Determining LLM performance across different languages is critical to ensure equitable healthcare delivery globally.

In this prospective observational study, we evaluate the performance of LLM-generated draft responses to patient messages across medical specialties in a major non-English academic medical center (14). We aim to investigate the adoption rate of these drafted replies by clinicians, assess their utilization and quality by comparing the initial drafts to the final sent replies, and analyze the time spent responding to patient messages. To our knowledge, this study is the first to explore this functionality in a non-English clinical setting, thus providing valuable insights into the applicability and potential benefits of LLMs beyond English-speaking contexts.

## Materials and methods

For this prospective observational cohort study all clinicians from 14 medical specialties of a large Dutch academic hospital were invited to use the LLM-generated draft replies after completing a mandatory e-learning. Clinicians who completed the mandatory e-learning were given access to the LLM-generated draft replies and were therefore included in the study. A brief presentation during clinician group meetings and walk-in hours was organized before and during the first weeks of the study. The study period lasted 16 weeks (March 1 to June 19, 2024).

### The use of large language models within the EHR

**Figure 1:**
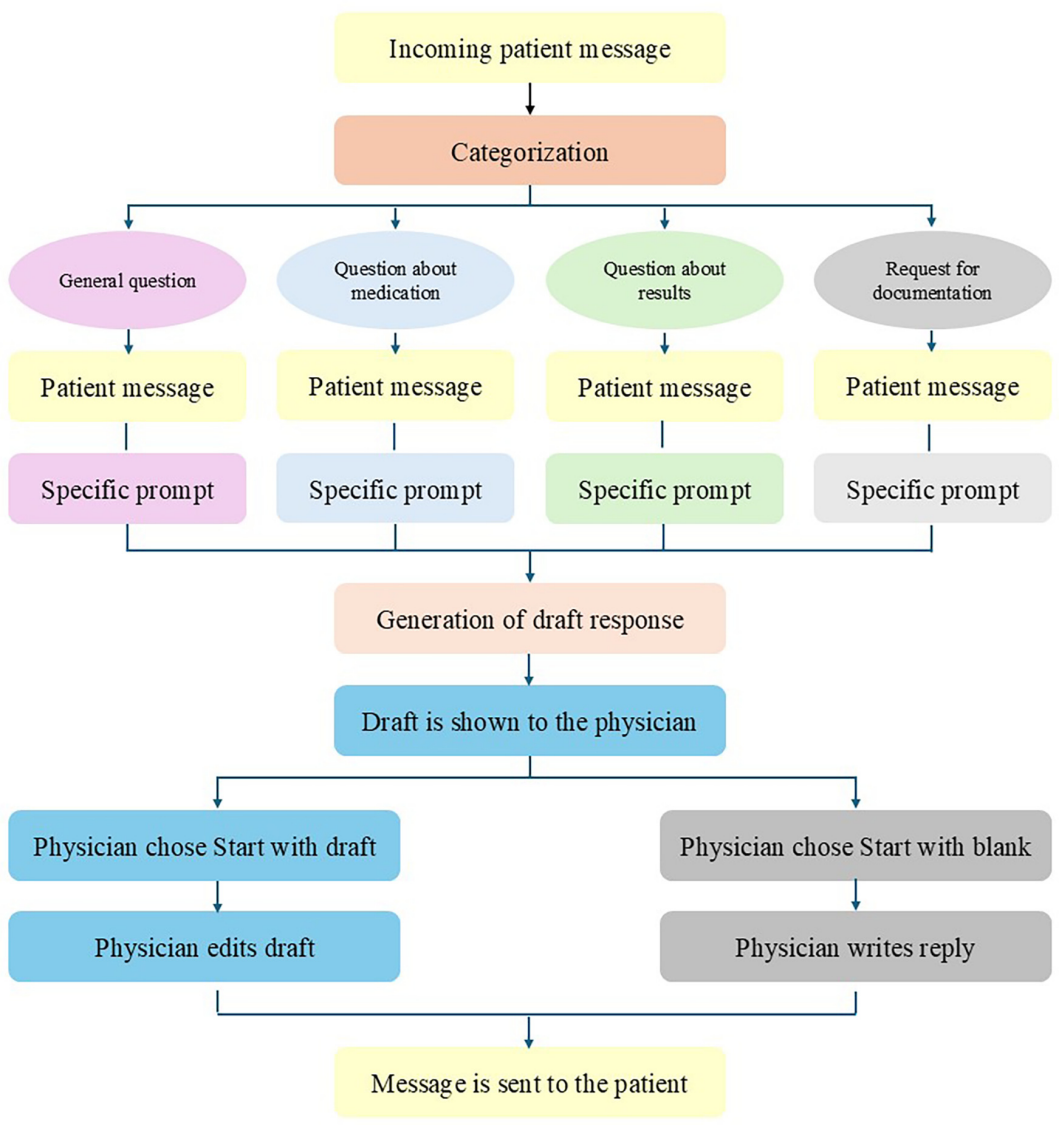
Flow of incoming patient messages, LLM integration, and the generation of draft replies.

LLM-generated draft responses to patient messages were generated using Microsoft's Azure Open AI through the EHR (Epic Systems Corporation, Verona, WI, USA). Incoming messages were automatically classified by the GPT-3.5 Turbo model into four categories (general, results, medications, and paperwork), based the contents of the message (Figure 1). These categories were chosen by our EHR provider Epic. The patient makes the choice by selecting the most appropriate category before sending the message. Each category had a different corresponding prompt, which included specific instructions, the patient message, and selected relevant information from the patient chart (e.g., name, age, allergies, medication list, future appointments, etc.). Embedded in the technical infrastructure of the EHR, the GPT-4 model was subsequently used to draft a response to the patient's message with a temperature setting of 0 for reproducibility purposes. Within the EHR, the clinician viewed both the patient message and the draft reply and was presented with the option to start with the draft or a blank reply. The clinician was not able to send the LLM-generated draft message immediately to the patient. This was done to ensure for safety. The review of the send message of clinician before sending is mandatory.

Prompt engineering (the act of writing sound instructions to the LLM) was performed by a team of multicenter medical and technical experts in an iterative manner (13, 14). Each iteration of the prompts was tested on at least 20 historical patient messages for each category. Clinicians that are part of the hospital's AI team provided feedback on correctness, harmfulness and usefulness to prompt engineering team. After 3–6 iterations, depending on the category, and when the clinicians deemed the output sufficient, the final iterations of the prompt were used for this study. An independent group of six physicians then extensively tested the functionality with the final for reliability during a 3-months pilot phase in an electronic dummy environment to make sure the model's responses were adequate. The prompts were then fixed at deployment. The clinicians did not have access to the prompts. The same prompts of each category were used for all clinicians, regardless of medical specialty. Due to an agreement with Epic, we are restricted from publishing these prompts.

### Outcome measures

To investigate the adoption and use of these LLM-generated draft replies, we assessed the rate of use of the draft responses (e.g., starting with the draft vs. starting with a blank response), the level of adjustments to the drafted responses and the time spent on these adjustments, as well as read time and total time spent in inbox.

To objectively evaluate the difference between the original draft reply and the final message sent to the patient, the validated ROUGE-1 and BLEU-1 scores for analysis of computational linguistics and natural language processing were used (15–17). The ROUGE-1 score measures the overlap of n-grams [contiguous sequence of n items (words) from a given sample of text] between the generated draft message and the final message sent and is recall oriented. The BLEU-1 score measures the precision of n-grams, comparing an index text to a reference text at the level of individual words (precision oriented). In this study, n-grams of 1 was used (ROUGE-1 and BLEU-1). Higher ROUGE-1 and BLEU-1 scores reflect textual similarity on word-level matching and thus indicate fewer adjustments made by clinicians to the initial LLM-generated draft responses. As clinicians could have inadvertently started with the draft while they intended to start with a blank response, thus removing or adjusting the draft message almost entirely, we performed sub-analyses using a BLEU-1 cutoff of 0.1 (precision oriented) or more to identify these instances. A BLEU-1 score of less than 0.1 indicates that the two compared texts have a less than 10% word match. This threshold was chosen based on an analysis of the median and interquartile range of the BLEU-1 scores in the dataset. In addition, empirically, negligible unigram overlap (<10%) indicates random or non-meaningful similarity in NLP evaluations.

To analyze the time used to either start a blank or adjust the drafted reply, we measured three time intervals (in seconds):

• Read to action time: from last message view to reply start;
• Drafting time: from reply start to send;
• Total time: from last view to send.

These time intervals were measured using audit-log data. Messages with read-to-action times above 10 min were excluded (4%), as were messages with patient draft times above 6 min (4%). These thresholds were chosen to exclude outliers where extended durations likely reflected interruptions or multitasking, making such cases less representative of routine drafting behavior. This approach ensured that the measured times reflected the actual drafting and replying times more accurately.

### Statistical analysis

SPSS ® Statistics version 28.0 (Armonk, NY: IBM Corp.) was used to analyze the data. Descriptive statistics for baseline characteristics are presented depending on the normality of their distribution. For comparison, Student's t-tests, the Mann–Whitney *U* test, or the *χ*^2^ test were used, depending on the type of variable studied. All studied time variables were non-normally distributed. Pearson's correlation coefficient was used for correlations. Statistical significance was set at *P* < 0.05. For natural language processing analyses (baseline characteristics, ROUGE, and BLEU-1 scores), Python (version 3.12.2) was used [Natural Language Toolkit (NLTK) packages]. NLTK scores are presented as percentages and are designed to compare two sets of texts. The ROUGE-1 recall represents the percentage of words that match between the LLM-generated draft reply and the final message sent (where 100 represents two equal texts). The BLEU-1 score reflects the number of similar words divided by the total words (as a percentage), with 100 representing two equal texts.

### Privacy and ethical considerations

All LLM interactions are managed by the EHR vendor under existing strict contractual agreements. Human review by third parties, such as Microsoft, is disabled. All patient-sensitive data are encrypted in transit to and from the LLM. Decrypted incoming and response messages remain within the hospital's digital infrastructure and cannot be accessed by third parties.

This study was prospectively registered (no. 19035). Our institutional review board granted permission. The functionality of generating draft responses to patient messages using generative AI is intended to reduce the administrative burden, in line with European regulations. The AI-tool described in this study was used according to the Declaration of Helsinki (ref. M24.328217) study and does not fall within the scope of the Medical Device Regulation as it is not intended for a medical purpose and does not provide clinical decision support. The generated output is always reviewed by the responsible clinician, using the ‘human in the loop’ principles.

## Results

**Figure 2:**
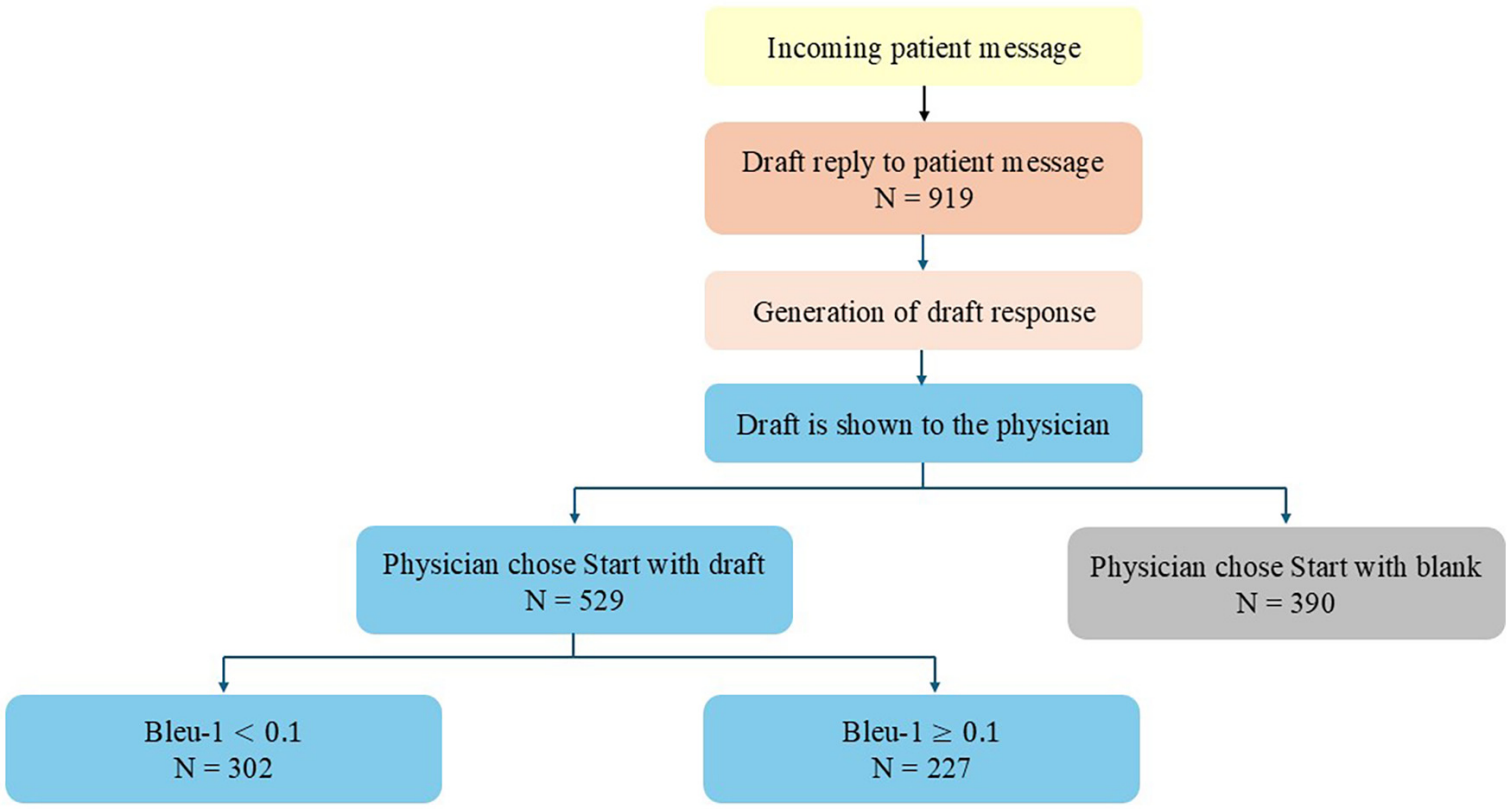
Flowchart of messages answered with draft replies.

During the study period of 16 weeks, a total of 919 messages were sent to patients by 100 clinicians from 14 different medical specialties participated in the study. Clinicians chose to use the drafted response as a template for the sent answer in 58% of the cases (*n* = 529), and 42% (*n* = 390) used a blank reply (Figure 2). When the draft responses were used, 43% (*n* = 227) used more than 10% of the suggested text in their final response.

An example (translated from Dutch to English for the purposes of this manuscript) of a patient message, suggested draft reply, and sent message:

**Table:** 

*Suggested draft reply*	*Send reply*
It is correct that the Norovirus can remain in the stool for up to three weeks after the symptoms. It would be best to send the stool after this period has passed to get the most accurate results.	How annoying that you have had stomach flu. Hopefully you have recovered a bit. The Noro virus is indeed contagious for 3 weeks. I see that you are going to send your stool in calprotetctin. I would advise you to do this 4 weeks after the virus. If this value is high, we can consider using additional cultures (for viruses/bacteria). I hope to have informed you sufficiently. Get well soon! And if there are any questions, please let me know.

When we began this study, we anticipated translation issues due to the Dutch-speaking environment. However, the problem was less significant than expected. The mistakes that did occur—such as translating ‘hearing loss’ as ‘no ears’—were easily corrected during the prompting phase. After these initial corrections, translation issues were no longer a problem for the study.

### Natural language processing metrics

The median ROUGE-1 and BLEU-1 scores are presented in Table 1. ROUGE-1 score was 0.86 for the drafted messages with 10% or more match between the draft and final message (BLEU-1 score ≥0.1), compared to 0.58 for drafted messages with less than 10% similarity (BLEU-1 score <0.1, *p* < 0.001). For blank replies, the median ROUGE-1 score was 0.16, and the median BLEU-1 score was 0.01. The percentage of words adjusted for drafted messages with BLEU-1 ≥ 0.1 was 18% and 51% for BLEU-1 scores <0.1 (*p* < 0.001). When starting with a blank reply, 46% were adjusted compared to the drafted message.

**Table 1:** ROUGE-1 and BLEU-1 scores, word counts, and percentage of draft text retained across reply types.

Category	Number of messages	ROUGE-1	BLEU-1	Words adjusted (%)^a^	Word count drafted reply	Word count sent reply
Blank	390	0.16 [0.12]	0.01 [0.01]	46 [49]	49 [27]	34 [29]
Drafted	529					
<10% match	302	0.58 [0.27]	0.01 [0.03]	51 [51]	50 [28]	37 [41]
≥10% match	227	0.86 [0.28]	0.51 [0.41]	18 [29]	53 [36]	54 [38]
Short	53	0.85 [0.29]	0.61 [0.46]	18 [33]	34 [9]	35 [14]
Middle	117	0.87 [0.32]	0.45 [0.39]	20 [31]	53 [21]	53 [26]
Long	57	0.83 [0.26]	0.58 [0.32]	18 [26]	100 [20]	91 [42]

Data provided in Median [IQR].

Length of message (words) based on quartiles (Q):

Short: 15–42 (Q1); Middle: 43–78 (Q2 & Q3); Long: >78 (Q4).

^a^ The difference between the number of words of the suggested draft reply and the sent reply was divided by the number of words of the suggested reply, times 100%.

### Time metrics

Read-to-action times did not differ between heavy (≥10%) and light (<10%) draft users (53 s vs. 54 s; *p* = 0.280; Table 2). Clinicians who discarded most of the draft (<10% retained) spent more time drafting (100 s vs. 74 s; *p* = 0.001). The total response times were similar across groups.

**Table 2:** Time metrics.

Category	Read-to-action (s) median [IQR]	Patient draft time (s) median [IQR]	Total time (s) median [IQR]
Blank reply	34 [12–96]	88 [51–156]	157 [86–265]
Drafted reply			
<10% match	54 [25–101]	100 [48–172]	171 [106–267]
>10% match	53 [32–107]	74 [37–141]	153 [85–254]
Short	56 [29–128]	43 [24–75]	117 [64–184]
Middle	53 [29–103]	74 [40–124]	144 [83–254]
Long	55 [39–105]	145 [77–213]	198 [150–313]

s, seconds. *p*-values in text.

Read-to-action time was faster for users starting a blank reply (34 vs. 53 s, *p* < 0.001), whereas patient draft time was longer compared to clinicians using drafted replies with ≥10% match (88 vs. 74 s, *p* = 0.023). The total time was not significantly different (*p* = 0.692). When the reply is started with a blank message or a drafted message with less than 10% match, read-to-action is faster (44 vs. 53 s, *p* < 0.001), patient draft time is longer (93 vs. 74 s, *p* = 0.003), and total time is not significantly different compared to using a drafted reply with ≥10% similarity (162 vs. 153 s, *p* = 0.244). The duration of the patient draft time increased accordingly with the message length (R = 0.569, *p* < 0.001, where R represents the correlation coefficient).

### Adoption between medical specialties

There was a large variation in the adoption of the LLM-drafted responses between medical specialties (Table 3).

**Table 3:** Adoption of LLM drafted replies for the different medical specialties.

Department	Start with draft		Majority of the draft text used	Total time (s)	ROUGE-1	BLEU-1	Start blank	
	Number	%	%	Seconds	Score	Score	Number	%
Dermatology	25	68	48	117 [84]	0.8 [0.3]	0.3 [0.5]	12	32
Gastro-Enterology	50	30	50	151 [169]	0.9 [0.2]	0.6 [0.4]	118	70
Gynecology	75	69	27	312 [149]	0.7 [0.3]	0.4 [0.5]	33	31
Nephrology	30	44	67	138 [236]	0.9 [0.3]	0.7 [0.5]	38	56
Neurology	73	95	63	111 [173]	0.9 [0.3]	0.6 [0.5]	4	5
Oncology	15	21	53	161 [240]	1.0 [0.2]	0.4 [0.5]	55	79
Otolaryngology	88	93	43	133 [163]	0.9 [0.3]	0.4 [0.4]	7	7
Orthopedics	27	100	41	157 [235]	0.9 [0.3]	0.6 [0.6]	0	0
Rheumatology & Immunology	119	50	32	188 [244]	0.8 [0.3]	0.5 [0.5]	121	50
Total	**502**	**63**	**47**	**163** [188]	**0.9 [0.3]**	**0.5 [0.4]**	**390**	**42**
Medical specialties with low number of messages therefore no statistics are performed.								
Endocrinology	11	92	36				1	8
Ophthalmology	3	100	50				0	0
Pediatric Cardiology	6	100	0				0	0
Pediatric Nephrology	4	80	75				1	20
Psychiatry	3	100	0				0	0

s, seconds. ^a^BLEU-1 score ≥0.1; NA, not applicable.

## Discussion

In this prospective observational study, we evaluated the effect of integrating an LLM into draft replies to patient messages with regard to adoption, quality, and potential time savings for clinicians. This study was the first to evaluate this functionality in a non-English hospital setting.

Our findings showed that clinicians chose to use the draft response as a template in the majority of cases (58%). Of the physicians who used more than 10% of the drafted message (43%), the ROUGE-1 score was high (0.86), meaning that the clinician used 86% of the LLM-generated draft response in their final message to the patient. Furthermore, in this group, the draft time was significantly shorter than when the clinician started with a blank reply or removed >90% of the generated text (BLEU-1 < 0.1 group). These findings indicate that when the draft is deemed appropriate to use as a template by the clinician, the LLM-generated drafts are considered useful, which also translates into shorter draft times. Despite shorter drafting times when using the draft, higher read-to-action times offset these gains, resulting in similar overall time spent in inbox.

Despite the absence of significant time savings, the use of LLM-generated draft replies in 58% of the messages suggests that clinicians may perceive benefits beyond efficiency. Possible explanations include cognitive offloading, more structured or articulate phrasing, or perceived improvements in tone and empathy. Moreover, even if the total time remains unchanged, the mental effort required to begin a response may be lower with a draft. These aspects were not directly measured in our study but represent important areas for future investigation. Importantly, although all generated drafts were reviewed and edited by physicians, the potential for biased or misleading content from LLMs must be acknowledged. Ensuring clinician oversight (i.e., the ‘human in the loop’ principle) remains critical to safeguard patient communication.

In our study, LLM-assisted replies were approximately 60% longer than blank replies (54 vs. 34 words), depending on whether the clinician started with the draft or blank reply, respectively. Longer messages may convey greater empathy, detail, and reassurance, consistent with reports that AI-drafted replies are perceived as more compassionate and help reduce clinician cognitive load, which other studies have indicated (18, 19, 20). However, excessively long messages could risk overwhelming patients or diluting key information. The current study did not capture clinician or patient preferences regarding the optimal reply length, which indicates a gap for future research.

The use of the draft responses varied greatly between medical specialties, indicating that for some medical specialties, the draft responses are more useful than for others. Possible explanations include variability in message content complexity, differences in response workflows, and the degree to which the generic prompts are aligned with specialty-specific communication needs. For example, specialties with more procedural-type questions may have found the draft responses more applicable. Our study did not collect clinician perspectives on these factors. In accordance with Yalamanchili et al. (10), we advise future research to include structured surveys and semi-structured interviews to gather qualitative insights into specialty-specific barriers and facilitators. It is important to note that in our study, all medical specialties utilized the same prompts. Given the significant variation in adoption, creating specialty-specific prompts that incorporate relevant terminology and the most common query types for each field could enhance draft relevance and reduce the editing effort required by clinicians. We recommend that, in future research, specialty-specific prompts are utilized to assess their impact on adoption and message quality.

Tai-Seale et al. performed a similar study in which they measured read time, reply time, length of replies and physician likelihood to recommend LLM-generated drafts (9). Their study was performed with 52 primary care physicians, whereas our study was conducted in an academic hospital setting, and they compared baseline (before the feature was activated) with use after two time periods. They also used a contemporary control group as a comparator. A significant increase in read time and no significant change in reply time were reported; however, an in-depth analysis was not provided. For example, they did not indicate whether the LLM-generated draft was actually used or if the clinician started with a blank reply. Furthermore, the total time spent in inbox was not reported. Our study provides further analysis on the actual use and usefulness of LLM-generated draft replies, including how much of the draft Garcia et al. performed a before and after study, comparing the total time spent in inbox before and after the implementation of this technology together with a clinician survey (11). They reported no significant differences in read time or reply time but did note significant improvements in task load and emotional exhaustion reported by the clinicians. An in-depth analysis of the use of the LLM-generated drafts was not performed, but similar to our study, high variation in use across different medical specialties was reported.

Our study has several limitations that may have contributed to the moderate use of the initial LLM-generated draft responses. First, we did not evaluate clinician experience or trust and are therefore limited in explaining the reasons for their (lack of) use of the draft responses. Second, we used the same prompts for all medical specialties. However, different medical specialties require different knowledge and alternative ways of phrasing answers to patient questions. Using different prompts for various medical specialties could have significantly increased the quality of the draft responses. Third, we used a generic foundation model instead of a fine-tuned healthcare-specific model that was uniquely trained to draft responses to patient questions. Using a healthcare-specific model could significantly improve the quality of the output while being more time- and energy-efficient by requiring less computing power. Although current evidence suggests otherwise, a comparative study found that the performance of a fine-tuned healthcare-specific model was inferior to that of a foundation model for this specific task (21). It is anticipated that advancements in fine-tuning techniques will eventually yield models that surpass the performance of current foundation models for specific tasks. Finally, due to compliance with the European Medical Device Regulation, we specifically instructed the model not to provide any medical advice. However, as patient questions are highly likely to contain medical questions, this decision could have significantly affected the quality of the initial LLM-generated draft response from the clinician's perspective, thereby limiting the use of this technology in clinical practice. Fourth, we chose the ROUGE-1 and BLEU-1 metrics for evaluating text similarity because these metrics are widely used in natural language processing studies, making them well-recognized and validated tools for text evaluation. ROUGE-1 and BLEU-1 metrics are straightforward to implement, providing quick and efficient assessments of surface-level unigram overlap between texts. However, these metrics are insufficient for capturing synonyms, paraphrasing, and semantic nuances. While we acknowledge the potential benefits of more advanced metrics like ROUGE-L, BERTScore, Sentence-BERT, and BLEURT, we chose not to use these due to several limitations. The implementation of ROUGE-L is computationally intensive and complex. BERTScore and Sentence-BERT rely on transformer models that require significant computational and memory resources, making them less efficient for large-scale evaluation. Moreover, their performance depends heavily on the quality and diversity of their training data, which may not always capture specific nuances in medical communication.

Our study demonstrated that LLM-generated draft responses are moderately adopted by clinicians, with substantial variation across medical specialties. When used as templates, these drafts can reduce drafting times without compromising the final quality of the responses. However, the total time savings are offset by increased read times, suggesting that LLM tools require further refinement to balance efficiency and thoroughness. We recommend exploring specialty-specific prompts and fine-tuned healthcare-specific models to enhance adoption and usefulness. Future research should also focus on clinician and patient experience, task load, and trust in these systems to inform broader implementation strategies.

## Data Availability

The raw data supporting the conclusions of this article will be made available by the authors, without undue reservation.
